# The *Trypanosoma cruzi* Satellite DNA OligoC-TesT and *Trypanosoma cruzi* Kinetoplast DNA OligoC-TesT for Diagnosis of Chagas Disease: A Multi-cohort Comparative Evaluation Study

**DOI:** 10.1371/journal.pntd.0002633

**Published:** 2014-01-02

**Authors:** Koen De Winne, Philippe Büscher, Alejandro O. Luquetti, Suelene B. N. Tavares, Rodrigo A. Oliveira, Aldo Solari, Ines Zulantay, Werner Apt, Patricio Diosque, Mercedes Monje Rumi, Nuria Gironès, Manuel Fresno, Rogelio Lopez-Velez, José A. Perez-Molina, Begoña Monge-Maillo, Lineth Garcia, Stijn Deborggraeve

**Affiliations:** 1 Department of Biomedical Sciences, Parasite Diagnostics Unit, Institute of Tropical Medicine, Antwerp, Belgium; 2 Instituto de Patología Tropical e Saúde Pública and Hospital das Clínicas, Universidade Federal de Goiás, Goiania, Brazil; 3 Programa de Biología Celular y Molecular, ICBM, Facultad de Medicina, Universidad de Chile, Santiago, Chile; 4 Unidad de Epidemiología Molecular (UEM), Instituto de Patología Experimental, Consejo Nacional de Investigaciones Científicas y Técnicas (CONICET), Salta, Argentina; 5 Centro de Biología Molecular Severo Ochoa, Consejo Superior de Investigaciones Cientificas (CSIC)-Universidad Autónoma de Madrid (UAM), Cantoblanco, Madrid, Spain; 6 Tropical Medicine & Clinical Parasitology, Infectious Diseases Department, Ramón y Cajal Hospital, Madrid, Spain; 7 Facultad de Medicina, Universidad Mayor de San Simón, Cochabamba, Bolivia; Foundation for Innovative New Diagnostics (FIND), Switzerland

## Abstract

**Background:**

The *Trypanosoma cruzi* satellite DNA (satDNA) OligoC-TesT is a standardised PCR format for diagnosis of Chagas disease. The sensitivity of the test is lower for discrete typing unit (DTU) TcI than for TcII-VI and the test has not been evaluated in chronic Chagas disease patients.

**Methodology/Principal Findings:**

We developed a new prototype of the OligoC-TesT based on kinetoplast DNA (kDNA) detection. We evaluated the satDNA and kDNA OligoC-TesTs in a multi-cohort study with 187 chronic Chagas patients and 88 healthy endemic controls recruited in Argentina, Chile and Spain and 26 diseased non-endemic controls from D.R. Congo and Sudan. All specimens were tested in duplicate. The overall specificity in the controls was 99.1% (95% CI 95.2%–99.8%) for the satDNA OligoC-TesT and 97.4% (95% CI 92.6%–99.1%) for the kDNA OligoC-TesT. The overall sensitivity in the patients was 67.9% (95% CI 60.9%–74.2%) for the satDNA OligoC-TesT and 79.1% (95% CI 72.8%–84.4%) for the kDNA OligoC-Test.

**Conclusions/Significance:**

Specificities of the two *T. cruzi* OligoC-TesT prototypes are high on non-endemic and endemic controls. Sensitivities are moderate but significantly (*p* = 0.0004) higher for the kDNA OligoC-TesT compared to the satDNA OligoC-TesT.

## Author Summary

Accurate diagnosis of Chagas disease is challenging due to the latent character of the infection and the low parasite load in the blood. Molecular tests such as the polymerase chain reaction (PCR) detect the parasite's DNA and are generally very sensitive and specific. In this study we evaluated two prototypes of a standardized PCR diagnostic kit: the satellite DNA (satDNA) OligoC-TesT and the kinetoplast (kDNA) OligoC-TesT. Sensitivities and specificities of both tests were estimated in a multi-cohort phase II evaluation study with 187 chronic Chagas patients and 88 healthy endemic controls from Argentina, Chile and Spain, and 26 non-endemic controls from D.R. Congo and Sudan with potentially cross-reacting diseases. Specificities in the control persons were high (>97%) and the sensitivity of the kDNA OligoC-TesT (79.1%) was significantly higher than of the satDNA OligoC-Test (67.9%). In a next phase, the kDNA OligoC-TesT should be evaluated in specific niches where standard serological tools have their limitations, e.g. follow-up after treatment and diagnosing newborns and HIV co-infected patients.

## Introduction

*Trypanosoma (T.) cruzi* is a kinetoplastid protozoan parasite and the etiological agent of American trypanosomiasis or Chagas disease. An estimated 8 million people are infected and more than 25 million are at risk of contracting the infection [1]. Chagas disease is endemic in Latin America, but evolved to a global health problem due to migration to other continents [2]. *T. cruzi* has a broad host range including wild and domestic animals. The parasite is primarily transmitted by infected haematophagous *Triatominae* bugs. Additional transmission routes include blood transfusion, organ transplantation, congenital infection, accidental infection and consumption of contaminated food causing orally transmitted outbreaks [3]. The initial acute phase lasts 6–8 weeks and clinical symptoms are generally mild and non-specific. Also in the chronic phase, the majority of the infected individuals remain asymptomatic, but up to 30% develop cardiac and digestive complications that can be fatal [4]. *T. cruzi* is monophyletic, but genetically heterogeneous and divided in six discrete typing units (DTUs): *T. cruzi* I to VI. The different DTUs have been associated with specific geographical distribution, reservoirs, vectors, virulence, clinical manifestation and susceptibility to drugs [5].

Accurate diagnosis of Chagas disease is challenging due to the latent character of the infection [6]. The parasite load in the blood of acute phase patients is generally high enough to be detected by microscopic analysis of blood smears or buffy coat in microhaematocrit capillaries. However, only 1 to 2% of all infected individuals are actually diagnosed during this phase because of the non-specific clinical manifestations [7]. In chronic patients, the parasite load is often very low and diagnosis is mostly accomplished by serological methods such as the indirect immunofluorescence (IIF), indirect haemagglutination (IHA) and enzyme-linked immunosorbent assays (ELISA). Sensitivity of antibody detection tests is generally high, but false-positive results occur due to cross-reactions with antibodies induced by other microorganisms such as *Leishmania* spp. or *T. rangeli* [8]. *T. rangeli* is closely related to *T. cruzi* and is non-pathogenic to man but shares the same reservoir animals and vectors [9]. To increase specificity, it is recommended to subject a specimen to at least two different serological assays [7]. For post-treatment monitoring however, the long-term persistence of specific antibodies, even after successful treatment, makes serological tests less informative. In case of congenital infections, serology can only be used after 6 to 8 months of age, because of the presence of maternal antibodies in newborns [7].

The polymerase chain reaction (PCR) has been presented as a promising method for sensitive and specific detection of *T. cruzi* parasites, especially in newborns [10] and during follow-up after treatment [11]. Real-time PCR offers the possibility to quantify the bloodstream parasite load, which could be of particular interest to follow the response to trypanocidal drugs [12]. DNA targets that have been most widely used in diagnostic PCR's are the *T. cruzi* minicircle kinetoplast DNA (kDNA) and the 195-bp satellite DNA (satDNA) [13]. Recently, we reported the development of a PCR dipstick assay for standardised detection of *T. cruzi* DNA in biological samples [14]. This *T. cruzi* OligoC-TesT (Coris BioConcept, Gembloux, Belgium) targets the satDNA sequence [15]. PCR amplicons are visualised on a lateral flow device that contains internal controls for the PCR reaction and for the chromatographic migration. The phase I evaluation showed high sensitivity and specificity on a diverse panel of biological samples and indicated the potential of the *T. cruzi* OligoC-TesT as a molecular diagnostic tool for Chagas disease [14]. However, the analytical sensitivity of the *T. cruzi* OligoC-TesT was 100 to 1000 times lower for TcI compared to the other DTUs, which is probably due to the lower copy number of the satDNA in TcI [16].

A recent multicentre validation study assessed the diagnostic accuracy of 48 different PCR tests, including the *T. cruzi* OligoC-TesT. The study revealed highly heterogeneous results among the different PCR tests and a sensitivity of 72% (n = 32) and specificity of 60% (n = 10) of the *T. cruzi* satDNA OligoC-TesT [13].

To increase sensitivity without compromising on *T. rangeli* cross-reactivity, a new OligoC-TesT prototype targeting a conserved region of the *T. cruzi* minicircle kDNA was developed. To assess their diagnostic accuracy in different endemic regions, we evaluated both versions of the *T. cruzi* OligoC-TesTs in a multi-cohort phase II study with 187 Chagas patients and 114 controls.

## Methods

### Ethics

Ethical clearance for the study was obtained from the ethics committees of the Universidad de Chile, Fundacion Huesped (Argentina) and Hospital Universitario Ramón y Cajal (Madrid, Spain). Written informed consent was obtained from patients and from non-diseased persons. Adult participants provided their own consent and a parent or guardian provided consent for children. All samples were anonymized.

### DNA

Purified DNA from *T. cruzi* Cutia cl1 (TcI), TU18 cl93 (TcII), M5631 cl5 (TcIII), Dog Theis (TcIV), MN cl2 (TcV) and CL Brener (TcVI) was obtained from the DNA reference bank at the London School of Hygiene and Tropical Medicine (LSHTM, UK). DNA from *L. chagasi* and three *T. rangeli* isolates LEM2953, P13 and SJMC2 was obtained from the DNA reference bank at the Institute of Tropical Medicine Antwerp (ITM, Belgium). Concentrations were measured using the nanodrop ND-1000 UV-Vis spectrophotometer (NanoDrop Technologies, Wilmington, USA) and the DNA was stored at −20°C. To assess the lower detection limits of the *T. cruzi* OligoC-TesTs, tenfold serial dilutions of parasite DNA, ranging from 200 pg to 0.002 fg DNA per test reaction, were prepared in water (Accugene, Lonza, Belgium) containing 0.1 mg/ml bovine serum albumin (Promega, Madison, Wis.). Archived DNA from blood of diseased non-endemic control persons was obtained from the biobank at ITM: 15 confirmed visceral leishmaniasis patients from Sudan and 11 confirmed *gambiense* sleeping sickness patients from D.R. Congo.

### Study participants and reference tests

In this prospective study, participants were classified as healthy endemic controls and Chagas disease patients based on the results of the reference tests, as described below. Exclusion criteria for participation in the study were children below 12 years of age, serious illness and not signing the informed consent form.

*Healthy endemic controls* were recruited from the blood bank of the Clinical Hospital at the University of Chile in 2004 and 2011. Study participants were classified as healthy endemic controls if they had no history of Chagas disease, showed no clinical symptoms and their serum was negative in *T. cruzi* ELISA and IIF.

*Chagas disease patients* were recruited in the Santiago Metropolitan Region in Chile in 2009; in Salta province North-West Argentina between 2008 and 2010; and at the Hospital Ramón y Cajal in Madrid, Spain in 2011. The patients recruited in Madrid were immigrants originating from Bolivia. Study participants were classified as Chagas disease patients if positive test results were obtained in ELISA and IHA (Argentina) or ELISA and IIF (Spain and Chile). Specifications of the reference tests are given in table 1.

**Table 1:** Demographic characteristics of the participants recruited in the study and specifications of blood collections and reference tests.

Origin	Recruitment period	Participant groups	Number of participants	Age range	Median age	Ratio male∶female	% with Chagas clinical signs	Volume of blood collected (mL)	ELISA	IIF	IHA
Chile	2004 and 2011	Healthy endemic controls	88	Unknown	Unknown	Unknown	0	3.0	a	b	ND
	2009	Chagas patients	80	23–77	50.0	0.40	68	3.0	a	b	ND
Argentina	2008 to 2010	Chagas patients	73	18–77	50.0	0.78	43	5.0	c	ND	d
Spain	2011	Chagas patients	34	15–51	43.0	0.48	56	2.5	e	f	ND

Notes: ND: not done; a: ELISA Chagas III, GrupoBios S.A., Chile; b: In house protocol with *T. cruzi* Tulahuen strain; c: Chagatest ELISA recombinant v. 4.0, Wiener Lab, Argentina; d: Chagatest HAI, Wiener Lab, Argentina; e: In house protocol with *T. cruzi* Dm28, MC & T strain; f: In house protocol with *T. cruzi* Dm28,MC & T strain.

### Blood collection and DNA extraction

Blood was collected and instantly mixed with an equal volume of guanidium EDTA buffer (GEB; 6M guanidium chloride, 0.2 M EDTA, pH 8.0), stored at 4°C and 1 mL aliquots were shipped to ITM Antwerp, Belgium. Upon receipt, DNA was extracted in duplicate from 200 µL lysed blood using the QIAamp DNA blood mini kit (Qiagen, Hilden, Germany) for the cohort recruited in Chile in 2004 and the High Pure PCR Template Preparation Kit (Roche Applied Sciences, IN, USA) for the other blood specimens. DNA extractions were done according to the manufacturer's instructions but with some modifications for the High Pure PCR Template Preparation Kit. Briefly, 200 µL of the blood/GEB sample were mixed with 600 µL binding buffer, 100 µL proteinase K and 200 µL isopropanol. The mixture was loaded onto a filter tube pre-packed with glass fibers, followed by extensive washing as described in the kit's manual. Finally, the DNA was eluted with 200 µL elution buffer that was preheated at 70°C. DNA was stored at −20°C immediately after extraction.

### Index tests

*SatDNA OligoC-TesT*. The *T. cruzi* satDNA OligoC-TesT (Coris BioConcept) was performed as previously described by Deborggraeve et al. [14]. The OligoC-TesT kit contains all components needed for performing the PCR and the product analysis by dipstick. Briefly, sequences of the oligonucleotides were: Tc-Sat-F primer 5′CACTCTCTGTCAATGTCTGTTTGCGTG-3′ and Tc-Sat-R primer 5′-GAAATTCCTCCAAGCAGCGGATA-3′; *T. cruzi* detection probe 5′-TGGACACCAAACAACCC-3′; and internal control (IC) probe 5′-AGGGTCTACTGGGTTACCTG-3′. To the 47.3 µL satDNA Ampli-Mix (Coris BioConcept), 2.5 µL sample DNA and 1 unit Hot Star Taq polymerase (Qiagen) were added and the mixture was subjected to thermal cycling as follows: 94°C for 15 minutes, 40 cycles of 94°C for 20 seconds, 65°C for 20 seconds and 72°C for 20 seconds, and a final extension at 72°C for 1 minute. Forty µL denaturated amplification product were mixed with an equal volume of OligoStrip running buffer preheated at 55°C followed by dipping the *T.cruzi* satDNA Oligo-Strip into the solution. Test results were read qualitatively after 5 minutes, as previously described [14].

*kDNA OligoC-TesT*. The *T. cruzi* kDNA OligoC-Test (Coris BioConcept) is identical to the satDNA OligoC-TesT except for the following specifications. Primers target the conserved region of the *T. cruzi* minicircle and have the following sequences: TcK-F 5′-GTTTTGGGAGGGGCGTTCAA-3′ and TcK-R 5′-TATATTACACCAACCCCAATCGAACC-3′. The detection probe was 5′-AAATAATGTACGGGGGAGATGCATG-3′ and the sequences of the IC and IC detection probe were identical as for the satDNA OligoC-TesT except for the IC that contained specific primer sites for TcK-F and TcK-R. Five µL sample DNA and 2 units of Hot Diamond Taq DNA polymerase (Eurogentec, Liège, Belgium) were added to 44.6 µL *T. cruzi* kDNA Ampli-mix (Coris BioConcept) and the reaction mixture was subjected to thermal cycling as follows: 95°C for 5 minutes, 40 cycles of 94°C for 20 seconds, 63°C for 20 seconds and 72°C for 20 seconds, and a final extension at 72°C for 1 minute. Forty µL denaturated amplification product were mixed with an equal volume of OligoStrip running buffer (Coris BioConcept) preheated at 55°C followed by dipping the *T.cruzi* kDNA Oligo-Strip into the solution. Test results were read after 10 minutes incubation. The executor of the index tests was one of the authors (KDW), a trained molecular biologist who was blinded to the results of the reference tests. The reference tests were performed at the sites of sample collection and the index tests were performed at ITM Antwerp.

### Statistical analysis

Sensitivities and specificities of the OligoC-TesTs were calculated from data entered into contingency tables. Differences in sensitivity and specificity between the two tests were estimated by the McNemar test. Repeatabilities of the tests were determined using the Kappa index. All calculations were estimated at a 95% confidence interval (95% CI) using Wilson's score.

## Results

### Study participants

We recruited 88 healthy endemic controls and 187 Chagas patients between 2009 and 2012. Demographic characteristics of the study participants are presented in table 1. Time between sample collection and conducting the index tests was maximum six months.

### Analytical sensitivity and specificity

**Figure 1:**
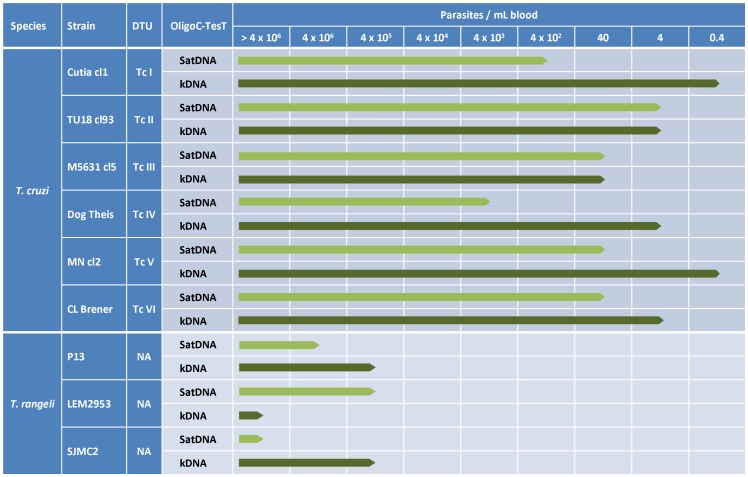
Analytical sensitivity and analytical specificity of the *T. cruzi* satDNA OligoC-TesT and kDNA OligoC-TesT on DNA from 6 *T. cruzi* and 3 *T. rangeli* reference strains. DTU = discrete typing unit, NA = not applicable.

The analytical sensitivity and specificity of the *T. cruzi* satDNA and kDNA OligoC TesTs were evaluated on tenfold serial dilutions of DNA from reference *T. cruzi* strains representing the 6 DTUs and from 3 *T. rangeli* strains. Serial dilutions were prepared in duplicate. Considering that one parasite contains about 100 fg of DNA and taking into account the dilution factors during DNA extraction (800× for the satDNA OligoC TesT and 400× for the kDNA OligoC TesT), the detection limit of each test was expressed as the number of parasites per mL of blood that yielded a positive signal in both repetitions. An overview of the analytical sensitivities and specificities of the two OligoC-TesTs is presented in figure 1. Generally, the OligoC-TesT targeting the kDNA showed a higher analytical sensitivity (lower detection limit: 0.4 to 40 parasites/ml blood) than the OligoC-TesT targeting the satDNA (lower detection limit: 4 to 4000 parasites/ml blood). On the serial dilutions of the *T. rangeli* DNA the two tests showed lower detection limits of 400,000 parasites per mL of blood or higher. No cross-reaction of the satDNA OligoC-TesT with *Leishmania* DNA was observed in our proof-of-concept study reported in 2009 [14]. In the current study, we analyzed 5 ng *L. chagasi* DNA with the kDNA OligoC-TesT and the test remained negative.

### Diagnostic accuracy (table 2)

**Table 2:** Sensitivities and specificities of the *T. cruzi* satDNA and kDNA OligoC-TesTs on 187 Chagas patients and 114 endemic and non-endemic controls from Chile, Argentina, Spain, Sudan and D.R. Congo.

				SatDNA OligoC-TesT				kDNA OligoC-TesT			
Origin	Number of specimens per participant group			Sensitivity % (95% CI)		Specificity % (95% CI)		Sensitivity % (95% CI)		Specificity % (95% CI)	
	Diseased non-endemic controls	Healthy endemic controls	Chagas disease patients	I	II	I	II	I	II	I	II
Sudan	15					100 (79.6–100)	100 (79.6–100)			100 (79.6–100)	100 (79.6–100)
D.R. Congo	11					100 (74.1–100)	100 (74.1–100)			100 (74.1–100)	100 (74.1–100)
Chile		88	80	78.6 (68.6–86.3)	67.5 (56.6–76.8)	98.9 (93.8–99.8)	94.3 (87.4–97.6)	93.8 (86.2–97.3)	91.3 (83.0–95.7)	96.6 (90.5–98.8)	98.7 (93.8–99.8)
Argentina			73	49.3 (38.2–60.5)	53.4 (42.1–64.4)			63.0 (51.6–73.2)	52.1 (40.8–63.1)		
Spain^a^			34	82.4 (66.5–91.7)	58.8 (42.2–73.6)			79.4 (63.2–89.7)	76.5 (60.0–87.6)		

Notes: 95% CI = 95% confidence interval; Non-end. = Non-endemic; I = first repetition; II = second repetition; N.D. = not done due to limited number of test kits available;

^a^ All Chagas patients recruited at the Hospital Ramón y Cajal in Madrid were from Bolivian origin.

Sensitivities, specificities and repeatability of the *T. cruzi* satDNA and kDNA OligoC-TesTs were calculated on the 26 diseased non-endemic controls, 88 healthy endemic controls and 187 Chagas patients.

Both OligoC-TesTs showed high specificity on the diseased non-endemic and healthy endemic controls, ranging from 94.3% to 100%. The overall specificities in these participant groups were 99.1% (95% CI 95.2%–99.8%) for the satDNA OligoC-TesT and 97.4% (95% CI 92.6%–99.1%) for the kDNA OligoC-TesT. No significant difference in specificity was observed between the two tests (*p* = 0.317).

Considering all Chagas patients recruited in the study, the kDNA OligoC-TesT showed a significantly higher sensitivity (79.1%, 95% CI 72.8%–84.4%) than the satDNA OligoC-TesT (67.9%, 95% CI 60.9%–74.2%) (*p* = 0.0004). Sensitivities of the OligoC-TesTs on the Chagas patients from Argentina were generally low and ranged from 49.3 to 63.0%. The overall repeatability of the kDNA OligoC-TesT (kappa = 0.85, 95% CI: 0.74–0.96) was higher than of the satDNA OligoC-TesT (kappa = 0.66, 95% CI: 0.54–0.77).

## Discussion

Recently, the *T. cruzi* satDNA OligoC-TesT was developed as a standardised format for molecular detection of *T. cruzi* satDNA [14]. Here, we report on the development of a second prototype, the *T. cruzi* kDNA OligoC-TesT, and the phase II evaluation of both tests on human target populations in different endemic regions.

With representative strains of the 6 *T. cruzi* DTUs, we observed that the *T. cruzi* kDNA OligoC-TesT, detecting between 0.4 and 40 parasites/mL which corresponds with 0.02 to 2 fg DNA per µL, had a higher (DTU I, IV, V and VI) or equal (DTU II and III) analytical sensitivity than the *T. cruzi* satDNA OligoC-TesT. These values are comparable to the results obtained in the PCR standardisation study of Schijman et al. [13] wherein the authors reported detection limits between 0.01 and 10 fg/µL for the four best performing PCR's. Both OligoC-TesTs amplified purified *T. rangeli* DNA, but required between 10^2^ and 10^7^ times more template DNA than for *T. cruzi*. For the satDNA OligoC-TesT, this is probably due to the number of satDNA repeats being about 1000 times lower in *T. rangeli* than in *T. cruzi* [17]. For the kDNA OligoC-TesT, the higher detection limit for *T. rangeli*, was obtained by one base pair mismatch between the reverse primer and the kDNA sequence of *T. rangeli*.

The diagnostic specificity of the two OligoC-TesTs was 100% on diseased non-endemic controls (Sudan and D.R. Congo) and between 94% and 99% on healthy endemic controls (Chile). A systematic review of the diagnostic accuracy of PCR for chronic Chagas disease showed that most PCR evaluation studies found similar high specificities [18]. In a next step, prospective evaluation studies with consecutive enrolment of suspected cases should be conducted to have a more accurate estimation of the specificity. When considering all Chagas patients recruited in the study, sensitivities of the OligoC-TesTs were moderate (79.1% for the kDNA OligoC-TesT and 67.9% for the satDNA OligoC-TesT), but comparable with reported sensitivities for other *T. cruzi* PCR's applied on chronic Chagas patients. In the review cited above, sensitivities ranging from 50 to 90% were observed [18]. Similar sensitivities, from 63% to 69%, were reported for the four best performing molecular diagnostics in the multicentre validation study conducted by Schijman et al. [13]. Particularly during the chronic phase, *T. cruzi* parasites circulate in very low and variable numbers in the bloodstream [19], [20]. Therefore, although PCR appears to be a sensitive method for the detection of *T. cruzi* DNA, it may remain negative in patients with very low or intermittent parasitaemia. Recovering DNA from larger blood volumes than the 200 µl used in this study would enhance sensitivity. The sensitivities of the two OligoC-TesTs greatly varied between the different cohorts. We observed a lower sensitivity of the OligoC-TesTs in the patients from Argentina compared to the patients from Chile and Spain. All blood samples in the study were collected, stored and shipped following a standardized procedure. We checked for degradation of the DNA in the Argentinean blood samples using primers targeting the human cytochrome b gene [21]. All samples were positive in this control PCR indicating no DNA degradation (data not shown). Compared to the other cohorts, the Argentinean patients showed a lower percentage of patients with Chagas symptoms which may be related with parasite load. Recently, Moreira et al. estimated the parasite loads in the blood of chronic Chagas patients from Argentina, Brazil and Colombia [22]. The median parasite load in the patients from Argentina was 1.93 parasites per mL of blood which was lower than patients from Colombia but higher than from Brazil. Genetic differences of parasite strains and/or DTUs might influence parasitaemia in man and partially explain the discrepancies of PCR sensitivity between cohorts from different endemic areas. However, no association between DTU and parasitaemia in the blood of patients has been reported yet [23]. In addition, *T. cruzi* DTUs vary in DNA gene copy number, thus harbouring a variable number of repeats of the PCR targets. Elias et al. described that the satDNA sequences can be 4 to 6 times more abundant in one DTU compared to another [16]. Since DTU typing in the specimens in our study was not performed, no conclusions can be made regarding this aspect. In view of the high sensitivity and PCR dependence of the OligoC-TesTs it is essential to use standard precautions during collection of blood and DNA extractions to avoid cross contamination between samples, and to include sufficient negative controls to detect any such contamination.

The overall repeatability of the kDNA OligoC-TesT was good (kappa = 0.85) and higher than of the satDNA OligoC-TesT (kappa = 0.66). Repeatabilities of the OligoC-Tests are linked to the detection limit, thus sensitivity, which was also higher for the kDNA OligoC-TesT. Therefore, moderate repeatabilities might be caused by the target DNA content in the samples that is situated too close to the lower detection limits of the tests.

In conclusion, the diagnostic accuracies of two standardised PCR formats, the satDNA OligoC-TesT and the kDNA OligoC-TesT, were evaluated in a multi-cohort study on 301 persons from various endemic and non-endemic countries. The specificities of the two tests were high in non-endemic controls as well as healthy endemic controls. The kDNA OligoC-TesT prototype showed a significantly higher sensitivity compared to the satDNA OligoC-TesT. However, it is unlikely that the OligoC-TesTs will play a major role in the diagnosis of chronic Chagas disease because of their low sensitivity compared to standard serological tests. Furthermore, the OligoC-TesTs are restricted to laboratories with PCR facilities, trained personnel and infrastructures that reduce the risk of PCR contamination. Their impact outside the main health centers will thus be limited. In a next phase, the kDNA OligoC-TesT should be evaluated in specific niches where standard serological tools have their limitations, e.g. diagnosing newborns and HIV co-infected patients and follow-up after treatment.

## Supporting Information

Checklist S1 STARD checklist showing that all essential elements of a diagnostic evaluation study are included in the manuscript.
(PDF)

Figure S1 STARD flowchart describing the design of the study and the flow of the participants.
(PDF)
