# Supplementary figures and images for: The Trypanosoma cruzi Satellite DNA OligoC-TesT and Trypanosoma cruzi Kinetoplast DNA OligoC-TesT for Diagnosis of Chagas Disease: A Multi-cohort Comparative Evaluation Study
Source: PLoS Negl Trop Dis. 2014 Jan 2;8(1):e2633. doi: 10.1371/journal.pntd.0002633 (PMC3879245; doi:10.1371/journal.pntd.0002633)

General example

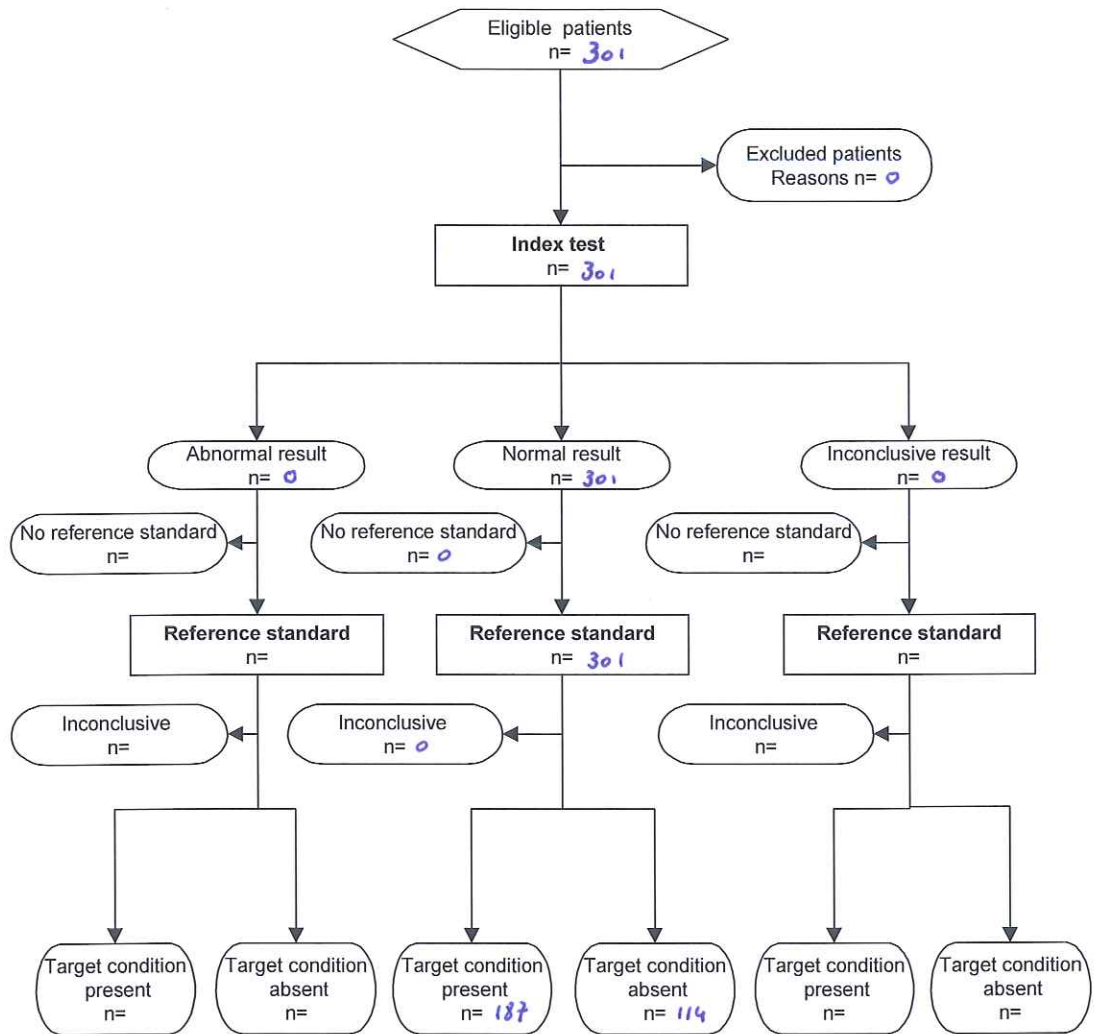

Supplement: Figure S1 — STARD flowchart describing the design of the study and the flow of the participants. (PDF) [file pntd.0002633.s002.pdf]
